# Climate-Driven Prediction of the Future Distribution of *Phytolacca americana* L. Using a BIOMOD2 Ensemble Modelling Framework

**DOI:** 10.3390/plants15111747

**Published:** 2026-06-04

**Authors:** Youning Wang, Chuan Du, Di Yang, Jiaxu Li, Wang Han, Liyan Zhao

**Affiliations:** 1 Hubei Key Laboratory of Resource Utilization and Quality Control of Characteristic Crops, Hubei Engineering University, Xiaogan 432000, China; wangyouning@hbeu.edu.cn (Y.W.);; 2 Agricultural Technology Extension Center of Jiangjin District, 278 Datong Road, Jijiang Subdistrict, Jiangjin District, Chongqing 402260, China; 3 College of Agriculture, Yangtze University, Jingzhou 434025, China

**Keywords:** *Phytolacca americana* L., invasive plant, species distribution model, BIOMOD2 ensemble, CMIP6, SSP scenarios

## Abstract

*Phytolacca americana* L. is an invasive perennial plant that has become increasingly widespread in China, but its current climatic suitability and future redistribution under climate change remain insufficiently quantified. This study aimed to identify the major environmental drivers of *P. americana* distribution and to project its potential habitat suitability under future climate scenarios. We compiled a national occurrence dataset and retained 683 quality-controlled presence records after taxonomic verification, coordinate checking, and 5 km spatial thinning. A BIOMOD2 ensemble modelling framework was used to integrate nine algorithms, and future projections were generated using CMIP6 climate data under SSP1-2.6, SSP2-4.5, SSP3-7.0, and SSP5-8.5 across four time periods from 2021 to 2100. The ensemble model showed strong predictive performance, with TSS = 0.804 and ROC = 0.967. May shortwave radiation, January mean temperature, and annual temperature range were identified as the dominant predictors of habitat suitability. Under current climate conditions, highly suitable habitats were mainly concentrated in warm and humid regions of eastern and southern China. Future projections indicated that suitable habitats may expand toward northern, northwestern, and higher-elevation regions, whereas highly suitable habitats may become redistributed or fragmented under stronger climate forcing. Centroid analyses further suggested non-linear, scenario-dependent shifts rather than a simple poleward expansion. These findings provide a spatial basis for early warning, targeted monitoring, and pathway-focused management of *P. americana* in China.

## 1. Introduction

Biological invasions are increasingly shaped by climate change and human-mediated dispersal. Rising temperatures, altered precipitation regimes, and more frequent extreme events can modify the environmental filters that determine establishment, persistence, and spread, while transportation networks and disturbed habitats facilitate propagule movement across regions [1,2,3,4,5,6,7,8]. For invasive plants, these processes may expand climatically suitable areas, alter invasion frontiers, and increase management pressure in agricultural and natural ecosystems [9,10,11,12,13,14].

*Phytolacca americana* L., a perennial herb native to North America, has become naturalized and increasingly widespread in China [15,16]. The species has high reproductive output, broad environmental tolerance, and bird-mediated seed dispersal, allowing it to colonize disturbed habitats, forest margins, road verges, abandoned land, and agricultural mosaics [15,16]. Its dense growth can suppress native vegetation, and its toxicity to humans and livestock further increases its ecological and agricultural relevance [16]. In China, its occurrence across multiple provinces suggests that both climatic suitability and human-mediated dispersal may contribute to its continued spread [15,16]. Therefore, identifying current and future climatically suitable regions is necessary for early warning, targeted monitoring, and regional invasion management.

Species distribution models (SDMs) provide a useful framework for linking occurrence records with environmental predictors and projecting potential habitat suitability under current and future climates [17,18,19,20]. However, predictions from a single algorithm may be sensitive to model assumptions, sampling bias, and extrapolation uncertainty [18,20]. Ensemble frameworks such as BIOMOD2 reduce algorithm-specific uncertainty by integrating multiple modelling approaches under a common calibration and evaluation workflow, and are therefore well suited for assessing the potential distribution of invasive species under climate-change scenarios [19,21,22,23]. Recent reviews also emphasize that human influence, land use, and dispersal pathways should be considered when interpreting SDM outputs in the Anthropocene [24].

Applying BIOMOD2 to invasive plants motivates several design choices that align with best practice. First, occurrence records for invasive species are often opportunistic and spatially clustered; BIOMOD2’s data-handling flexibility supports spatial thinning of occurrences and structured cross-validation (e.g., block cross-validation) to reduce over-optimistic performance and enhance geographic transferability [20]. Second, pseudo-absence or background sampling strongly influences calibration and discrimination. BIOMOD2 allows explicit prevalence control and multiple pseudo-absence strategies—including target-group and environmentally stratified designs—so that training data better represent available environments while limiting sampling bias [19]. Third, high collinearity among environmental predictors can obscure variable importance and inflate parameter variance. Predictor curation therefore combines ecological reasoning with correlation screening and variance-inflation controls to yield an interpretable set spanning thermal, hydro-meteorological, radiative, and topographic domains [19]. Fourth, projections to future climates require diagnostics for transferability. BIOMOD2 provides clamping and extrapolation flags, enabling qualification of predictions where environmental values move beyond the training domain and supporting conservative interpretation in novel climate space [18,19]. Finally, decision-support benefits from converting continuous suitability into threshold-based and consensus maps. BIOMOD2 supports threshold optimization (e.g., maximizing TSS) and the aggregation of agreement across algorithms and scenarios, allowing uncertainty to be communicated alongside suitability patterns [18,19].

Although SDMs have been widely used to evaluate invasive plant risks, China-scale assessments of *P. americana* remain limited, particularly those integrating multiple algorithms, curated occurrence records, seasonal climatic predictors, and multiple future SSP periods [15,16]. Moreover, previous studies have not consistently translated suitability projections into management-relevant information, such as priority regions for surveillance and potential dispersal corridors [14,19,24].

The present study adopts a BIOMOD2-based ensemble design for *P. americana* across China and, where relevant, adjacent regions that influence cross-boundary propagule pressure. Georeferenced occurrences are integrated with a curated suite of environmental predictors representing thermal, moisture, radiative, and topographic conditions. Within BIOMOD2, multiple algorithms are trained under repeated cross-validation, filtered using AUC, TSS, and Kappa, and assembled into a performance-weighted ensemble. Pseudo-absence strategies and prevalence settings are defined to balance calibration and discrimination, and spatial thinning with block cross-validation is used to mitigate sampling bias and improve transferability [19,20]. Projections are generated under CMIP6 scenarios (e.g., SSP1-2.6, SSP2-4.5, SSP3-7.0, SSP5-8.5) and multiple time slices (e.g., 2021–2040, 2041–2060, 2061–2080, 2081–2100), with clamping and extrapolation diagnostics reported to qualify transfer to novel climates [18,19]. Continuous suitability surfaces are converted to threshold-based and consensus maps that are suitable for surveillance design and risk communication [19].

Here, we developed a BIOMOD2-based ensemble SDM to evaluate the current and future potential distribution of *P. americana* in China. Specifically, we aimed to: (1) compile and filter a national occurrence dataset for *P. americana*; (2) identify the dominant environmental predictors associated with its potential distribution; (3) project habitat suitability under current climate and future CMIP6 SSP scenarios; and (4) identify regions where future monitoring and management should be prioritized. We hypothesized that seasonal thermal and radiative conditions would strongly shape current suitability, and that future warming would expand suitable areas toward northern and elevation-limited regions while potentially reconfiguring highly suitable habitats under stronger climate forcing. By linking ensemble SDM outputs with invasion-management needs, this study provides a spatial basis for early warning and targeted control of *P. americana* in China.

## 2. Materials and Methods

### 2.1. Data Sources and Preprocessing

#### 2.1.1. Occurrence Records

**Figure 1:**
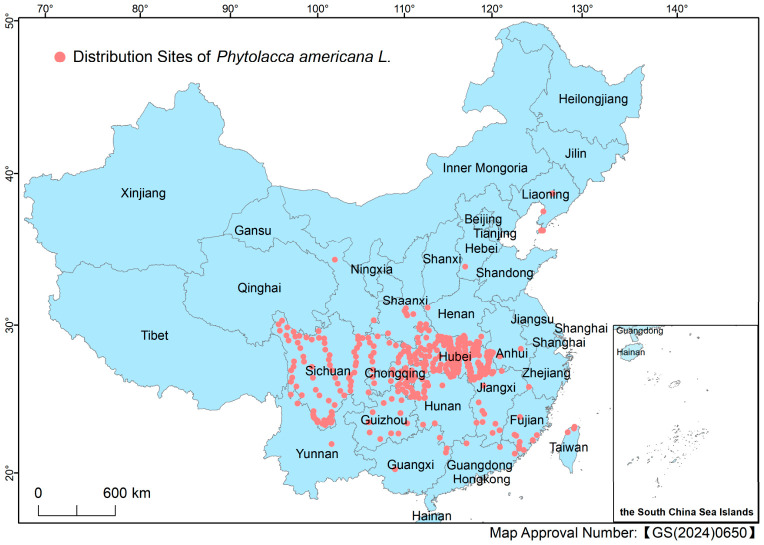
Distribution sites of *P american* in China after data cleaning and spatial filtering. Red dots represent the occurrence records used in the BIOMOD2 ensemble modelling.The dashed lines in the lower-right inset indicate the maritime boundary lines associated with the South China Sea Islands in the standard base map and are shown for cartographic reference only; they were not used as occurrence records or environmental predictors in the modelling analysis.

We compiled a national database of *P*. *americana* occurrences in China by integrating field surveys, herbarium repositories, and literature-derived records into a unified geospatial workflow (Figure 1). Field campaigns followed the Technical Regulation for the Census of Exotic Herbaceous Plants (NY/T 1861–2010) and used stratified random sampling in typical invasion habitats such as croplands and road verges [4], yielding 376 georeferenced presences. Herbarium data contributed 460 expert-verified records retrieved from the Chinese Virtual Herbarium (CVH), the National Specimen Information Infrastructure (NSII), the China Field Herbarium (CFH), and the Plant Photo Bank of China (PPBC) [25,26]. Literature screening added 26 occurrences from remote areas. Records underwent taxonomic validation and coordinate checks to remove errors and duplicates [17]. To mitigate spatial clustering and sampling bias, we applied a 5 km thinning radius in SDM Toolbox (ArcGIS 10.8), exported cleaned occurrences in CSV format, standardized coordinates to WGS84 (EPSG:4326), and annotated each record with latitude, longitude, elevation, and collection year [27]. After quality control, the final dataset comprised 683 high-quality presences aligned to the environmental raster grid.

#### 2.1.2. Environmental Predictors and Preparation

Current and future bioclimatic variables and topography were obtained from WorldClim and harmonized to a common spatial resolution and extent, with rasters exported in ASCII format for modelling [28]. Future climates were sourced from CMIP6 using the BCC-CSM2-MR general circulation model under SSP1-2.6, SSP2-4.5, SSP3-7.0, and SSP5-8.5 for four time slices (2021–2040, 2041–2060, 2061–2080, 2081–2100) [29,30]. Non-climatic predictors were held constant across scenarios to isolate climatic effects on potential suitability.

#### 2.1.3. Variable Screening

**Figure 2:**
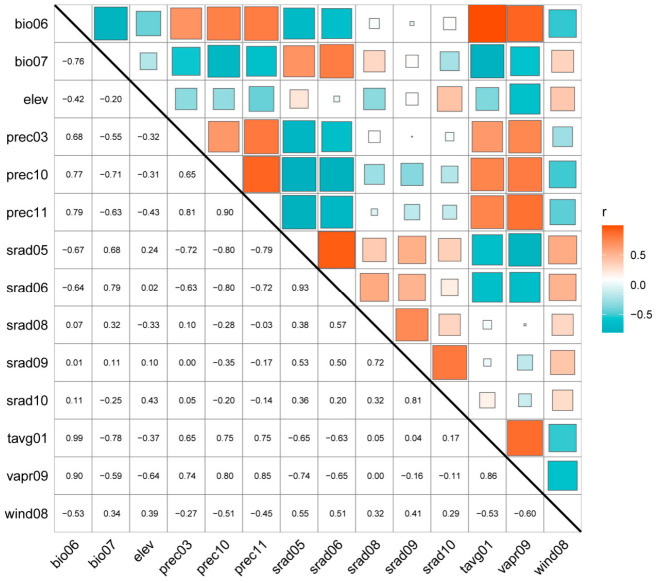
Correlation analysis of candidate environmental predictors for *P. americana* distribution modelling. Numbers in the lower triangle represent Pearson’s correlation coefficients (r), while the upper triangle visualizes the magnitude and direction of correlations. This analysis was used to identify collinearity among variables and to retain ecologically meaningful predictors for subsequent BIOMOD2 ensemble modelling.

An initial set of 104 environmental predictors was screened using a two-stage procedure that combined model-informed contribution filtering and collinearity control (Table 1). First, preliminary BIOMOD2 runs were conducted using all candidate predictors, and permutation importance was calculated for each variable. Variables with a mean importance value > 1% were retained as candidate predictors. This threshold was used as a preliminary filter to remove variables with negligible contribution while retaining predictors with potential ecological relevance. Second, Pearson correlation analysis was performed among the retained candidate variables. When two variables were strongly correlated (|r| ≥ 0.8), the variable with clearer ecological relevance to *P. americana* distribution or higher model contribution was retained. The final predictor set comprised nine variables representing thermal conditions (BIO07 and Tavg01), moisture availability (Prec03 and Prec10), solar radiation (Srad05, Srad08, and Srad10), wind condition (Wind08), and topographic context (Elev) (Figure 2).

### 2.2. BIOMOD2 Ensemble Modelling

#### 2.2.1. Algorithms and Model Setup

Species distribution modelling was conducted in R using BIOMOD2 to implement nine algorithms—GLM, GBM, GAM, CTA, ANN, FDA, MARS, RF, and MaxEnt—within a common modelling framework. Because only presence records were available for *P. americana*, pseudo-absence points were generated to represent the background environmental conditions available within the study area. Pseudo-absences were randomly sampled from raster grid cells where no confirmed occurrence records were present and were aligned with the same environmental raster resolution and extent as the occurrence data. Two independent pseudo-absence datasets of 1000 points each were generated. The number of pseudo-absences was selected because it exceeded the 683 retained presence records, provided broad coverage of available environmental space, and avoided excessive prevalence imbalance or unnecessary computational burden. For each algorithm, two independent modelling replicates were run, and each replicate used 75% of the data for model calibration and 25% for model evaluation.

#### 2.2.2. Pseudo-Absences, Replication, and Validation

Model performance was evaluated using the True Skill Statistic (TSS) and the area under the receiver operating characteristic curve (AUC). The two pseudo-absence datasets and two modelling replicates were used to reduce the influence of random pseudo-absence selection on model evaluation. Single-algorithm models with TSS > 0.70 were retained for ensemble modelling, because this threshold indicates good discriminatory performance and helps exclude poorly performing learners before ensemble construction. Replication across pseudo-absence sets and modelling runs allowed us to characterize performance variability among algorithms. Clamping and extrapolation diagnostics were inspected when transferring models to future climate scenarios to identify areas where environmental conditions exceeded the calibration range.

#### 2.2.3. Ensembling and Evaluation

Ensemble predictions were produced using weighted probability and relative majority procedures. TSS was used for ensemble weighting because it is insensitive to prevalence and balances sensitivity and specificity, making it suitable for presence–pseudo-absence species distribution modelling. For weighted probability ensembles, single-model outputs were combined using TSS-based weights normalized to sum to one, thereby emphasizing higher-performing base learners while maintaining algorithmic diversity [31]. For majority voting, binarized outputs were aggregated by relative majority at the grid-cell level. Ensemble performance was re-evaluated using TSS and AUC, and agreement layers were exported to facilitate communication of uncertainty.

### 2.3. Suitability Mapping and Classification

Continuous suitability outputs from BIOMOD2 (ASCII) were converted to rasters in ArcGIS and reclassified using Jenks’ Natural Breaks into four classes: unsuitable (0–0.25), low (0.25–0.50), moderate (0.50–0.75), and high (0.75–1.00) suitability (Table 2). This procedure maximizes between-class variance and minimizes within-class variance, yielding a standardized depiction of spatial heterogeneity. The resulting maps provide the basis for visualization and subsequent risk interpretation at management-relevant scales.

### 2.4. Quantification of Suitable Areas and Centroid Shifts

To quantify changes in habitat suitability, the classified raster outputs were used to calculate the area of each suitability class under the current climate and each future SSP/time slice. The area of each class was obtained by multiplying the number of raster cells in that class by the corresponding cell area and was summarized in km^2^. To quantify spatial redistribution, the centroid of suitable habitats was calculated for each scenario and period using the same classified suitability layers. Centroid displacement was expressed as geodesic distance from the current-climate centroid and as azimuth direction in degrees, measured clockwise from north. These quantitative outputs were used to complement the suitability maps and centroid-shift figures.

## 3. Results

### 3.1. Establishment of the BIOMOD2 Ensemble Model

Using curated occurrences of *P*. *americana* and 104 environmental predictors under contemporary climate, we implemented nine single-algorithm models within BIOMOD2, each run twice. Owing to distinct statistical foundations, single-model predictions differed, and evaluation metrics varied correspondingly. Based on S1, ROC indicated excellent discrimination for GLM (0.936), GBM (0.958), ANN (0.912), FDA (0.933), MARS (0.938), RF (0.968), and MaxEnt (0.904), with CTA (0.896) and SRE (0.814) reaching good levels. Kappa identified RF as best (0.806; the only “excellent”), followed by GBM (0.752), with all remaining models ≥ 0.60. TSS also ranked RF highest (0.848) and SRE lowest (0.627), with the other seven models attaining good performance. Applying a TSS > 0.70 filter, we excluded SRE and constructed an ensemble (EM) from the remaining algorithms. The EM achieved superior validation relative to its constituents, with TSS = 0.804 (95% CI: 0.783–0.825) and ROC = 0.967 (95% CI: 0.959–0.975), supporting high discriminatory capacity and robust species–environment signal.

### 3.2. Importance of Environmental Predictors

**Figure 3:**
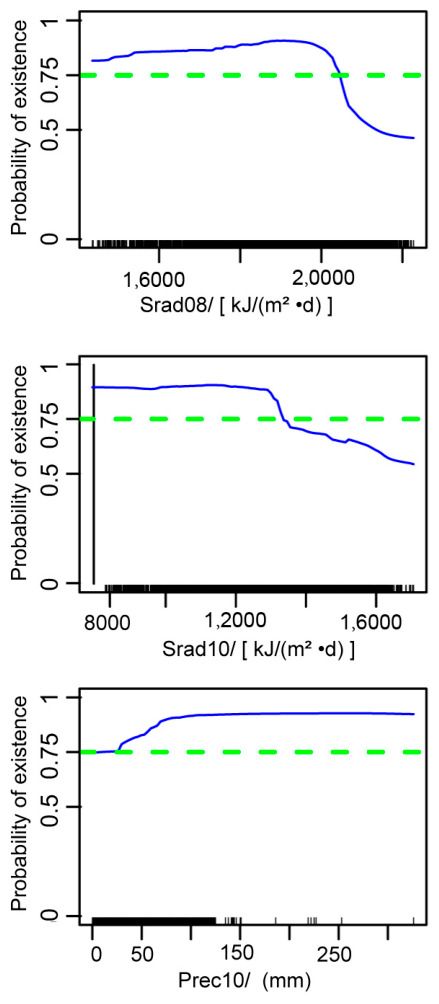
Partial response curves for key environmental predictors of *P. americana* distribution in the BIOMOD2 ensemble model. The blue curves represent the predicted probability of presence in response to changes in Srad08, Srad10, and Prec10, and the green dashed lines indicate the threshold value used for suitability interpretation. These response relationships suggest that the occurrence probability of *P. americana* is sensitive to seasonal variation in solar radiation and precipitation.

Because single-algorithm importance estimates differ, only well-performing models were considered when inferring drivers (Figure 3). BIOMOD2 does not return a unified importance profile for the ensemble; therefore, we applied a normalization approach to synthesize importance across retained models. The resulting pattern consistently highlighted May solar radiation (Srad05), January mean temperature (Tavg01), and temperature annual range (BIO07) as the most influential predictors, followed by October solar radiation (Srad10), October precipitation (Prec10), and August solar radiation (Srad08), with additional contributions from wind in August (Wind08) and elevation (Elev). These variables delineate thermal and radiative seasonality, moisture availability in autumn, and topographic context as key correlates of suitability.

### 3.3. Predicted Current Potential Distribution

**Figure 4:**
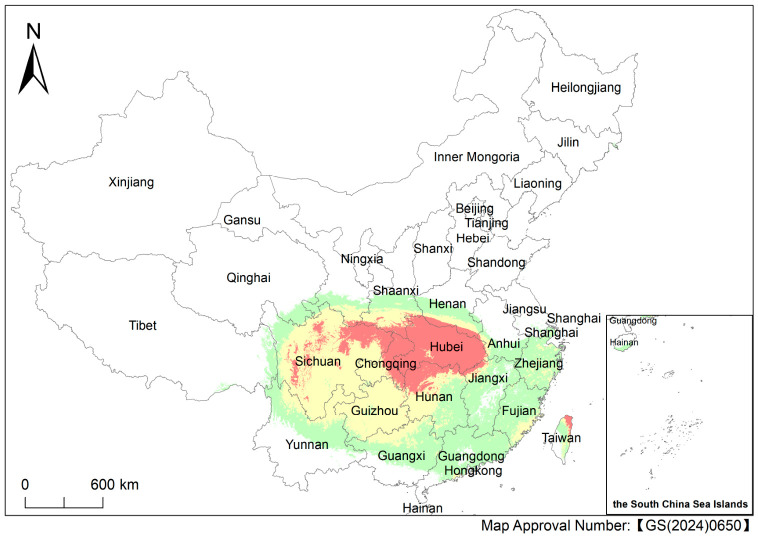
Spatial distribution of *P. americana* in China. Grey indicates unsuitable area, green indicates low suitability, yellow indicates moderate suitability, and red indicates high suitability. The predicted suitable habitats are mainly distributed in central, eastern, and southern China, with highly suitable areas concentrated in regions with favorable hydrothermal conditions.

Under present climate, suitability exhibits clear regional structure from high to low classes (Figure 4). High suitability (0.75–1.00) is concentrated across eastern and southern China, including parts of Shandong, Anhui, Zhejiang, Fujian, Henan, Hubei, Hunan, Jiangxi, Guangdong, Guangxi, and portions of Yunnan and Sichuan, where warm and humid conditions favor establishment. Moderate suitability (0.50–0.75) forms buffers around these cores, extending into sections of Hebei, Shanxi, Shaanxi, Guizhou, and Chongqing, reflecting partial climatic constraints. Low suitability (0.25–0.50) occurs at the periphery, including parts of Liaoning and Jilin, sectors of Gansu and Ningxia, and limited valleys in Tibet, where cold or arid conditions limit persistence. Unsuitable areas (0.00–0.25) dominate much of Xinjiang, Qinghai, large portions of Heilongjiang, and high-elevation zones of Tibet. The spatial pattern indicates potential pressure along northern and northwestern margins and at elevation-limited fronts should local thermal or moisture regimes become more permissive or human activities enhance propagule movement.

### 3.4. Predicted Future Potential Distribution Under SSP Scenarios

**Figure 5:**
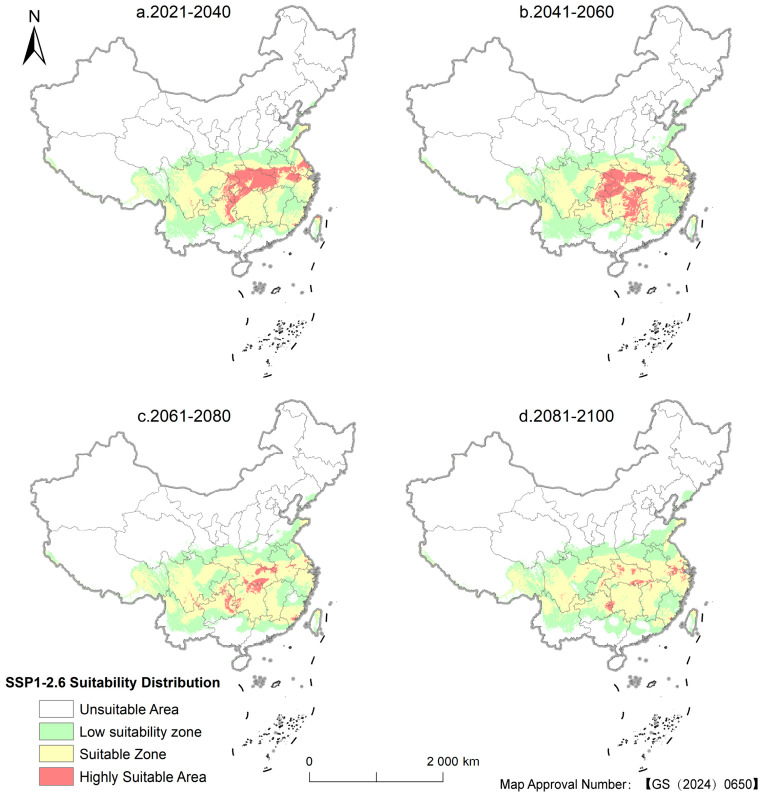
Spatial distribution of habitat suitability for *P. americana* in China under the SSP1-2.6 scenario during four future periods (2021–2040, 2041–2060, 2061–2080, and 2081–2100). Grey indicates unsuitable area, green indicates low suitability, yellow indicates suitable area, and red indicates highly suitable are.

**Figure 6:**
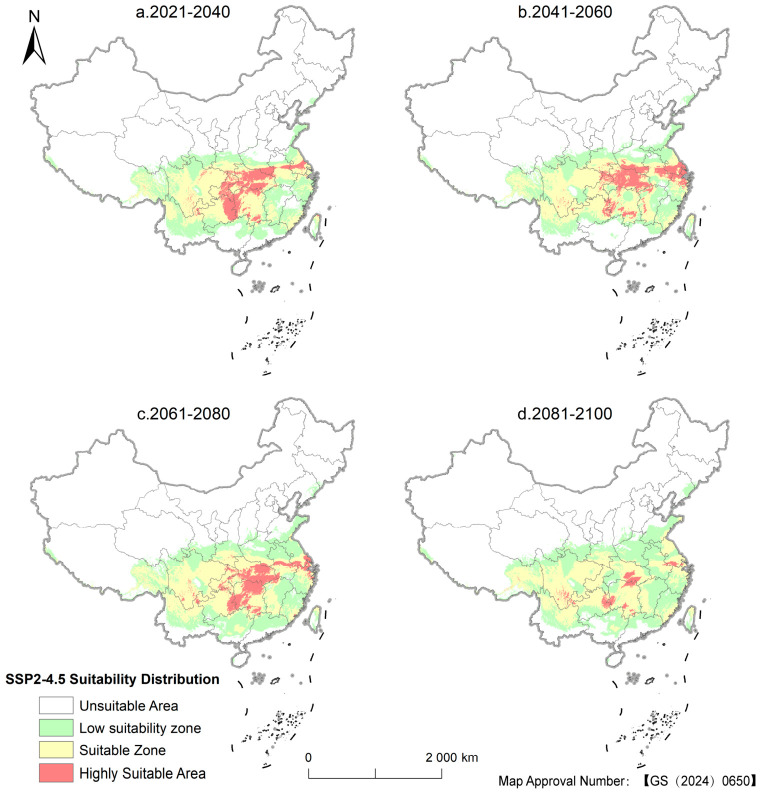
Spatial distribution of habitat suitability for *P. americana* in China under the SSP2-4.5 scenario during four future periods (2021–2040, 2041–2060, 2061–2080, and 2081–2100). Grey indicates unsuitable area, green indicates low suitability, yellow indicates suitable area, and red indicates highly suitable area.

**Figure 7:**
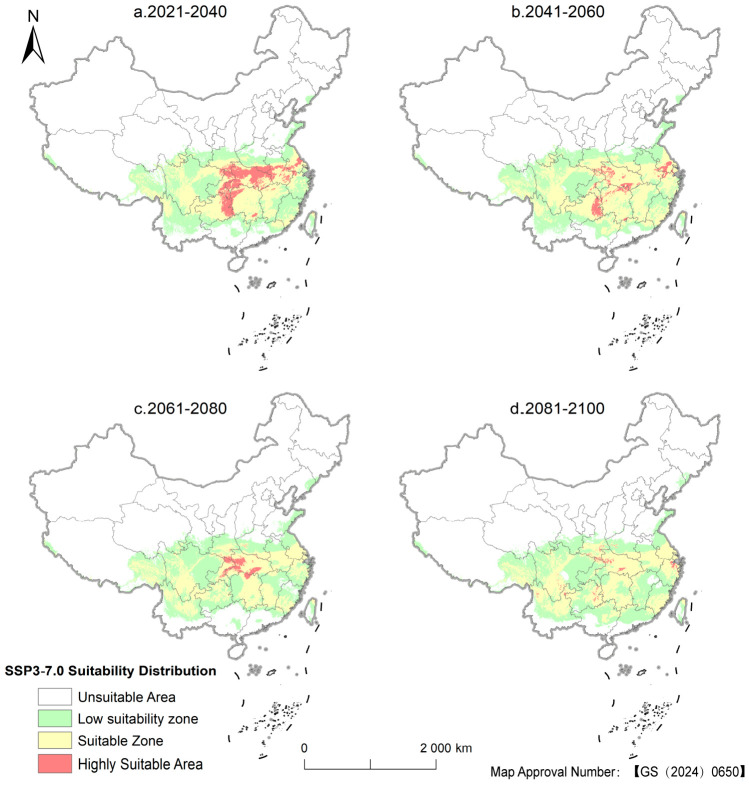
Spatial distribution of habitat suitability for *P. americana* in China under the SSP3-7.0 scenario during four future periods (2021–2040, 2041–2060, 2061–2080, and 2081–2100). Grey indicates unsuitable area, green indicates low suitability, yellow indicates suitable area, and red indicates highly suitable area.

**Figure 8:**
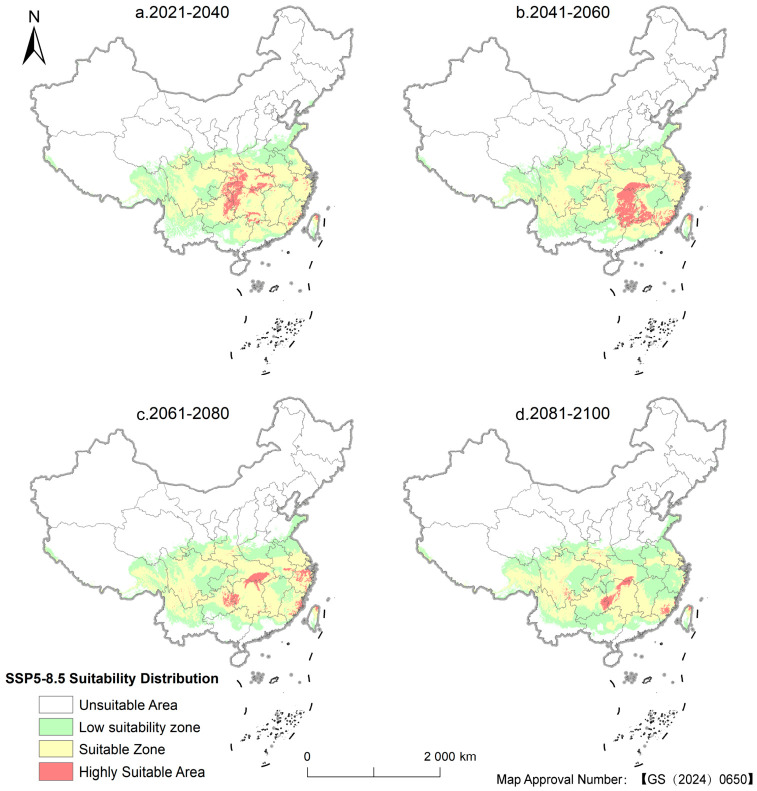
Spatial distribution of habitat suitability for *P. americana* in China under the SSP5-8.5 scenario during four future periods (2021–2040, 2041–2060, 2061–2080, and 2081–2100). Grey indicates unsuitable area, green indicates low suitability, yellow indicates suitable area, and red indicates highly suitable area.

**Figure 9:**
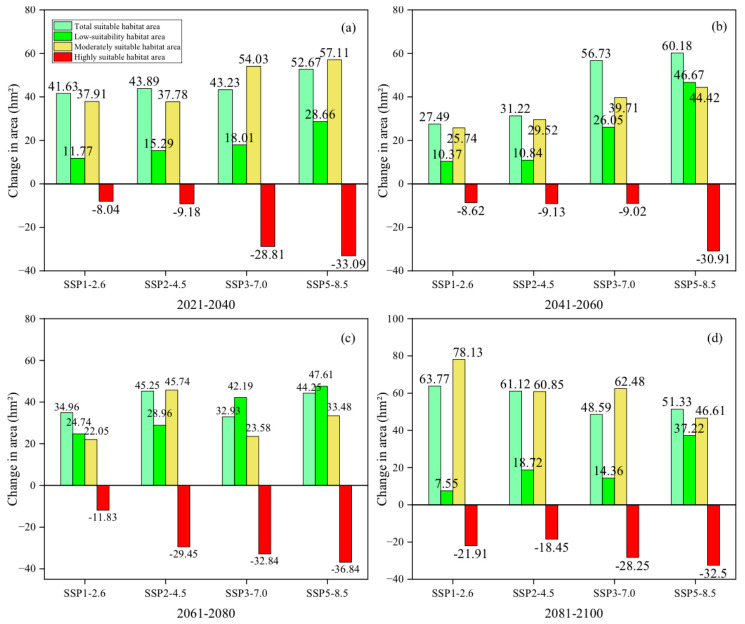
Changes in the area of suitable habitats for *P. americana* under future climate scenarios. (**a**) 2021–2040; (**b**) 2041–2060; (**c**) 2061–2080; (**d**) 2081–2100. Green, light green, yellow, and red bars represent changes in total, low-suitability, moderately suitable, and highly suitable habitat areas, respectively.

Across CMIP6 scenarios, suitability dynamics differed in both spatial extent and suitability class composition (Figure 5, Figure 6, Figure 7, Figure 8 and Figure 9). To complement the map-based interpretation, we quantified the area of unsuitable, low-suitability, moderate-suitability, and high-suitability habitats under the current climate and each future SSP/time slice. These area estimates show whether projected range changes mainly resulted from expansion of newly suitable areas, upgrading from low to moderate suitability, or redistribution of highly suitable habitats. Under SSP1-2.6, suitable habitats expanded progressively toward northern and northwestern margins, mainly through increases in low- and moderate-suitability areas. Under SSP2-4.5, the expansion became more evident during the mid- to late-century periods, with suitability gains in parts of northern China, loess and karst regions, and low-elevation valleys. Under SSP3-7.0, future suitability showed phased fluctuations, with early expansion followed by spatial consolidation or partial contraction in some marginal regions. Under SSP5-8.5, rapid early expansion was followed by stronger spatial reconfiguration, indicating that the total suitable area and the distribution of high-suitability habitats may respond differently under stronger climate forcing.

### 3.5. Future Shifts in Suitability Centroids

**Figure 10:**
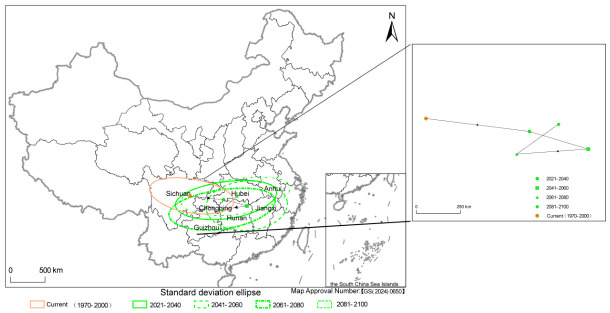
Standard deviation ellipses and centroid shifts of suitable habitats for *P. americana* under the SSP1-2.6 climate scenario. The ellipses represent the spatial distribution ranges of suitable habitats during the current period (1970–2000) and future periods (2021–2040, 2041–2060, 2061–2080, and 2081–2100). The inset shows the migration trajectory of the habitat centroid across different periods.

**Figure 11:**
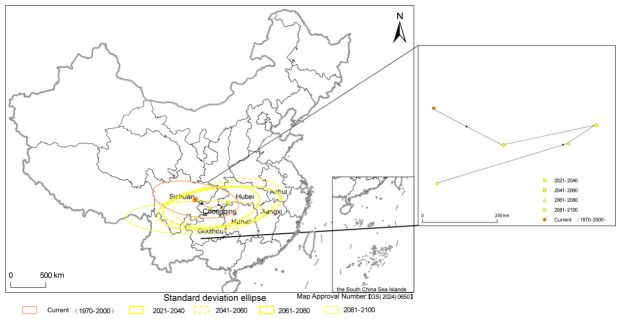
Standard deviation ellipses and centroid shifts of suitable habitats for *P. americana* under the SSP2-4.5 climate scenario. The ellipses represent the spatial distribution ranges of suitable habitats during the current period (1970–2000) and future periods (2021–2040, 2041–2060, 2061–2080, and 2081–2100). The inset shows the migration trajectory of the habitat centroid across different periods.

**Figure 12:**
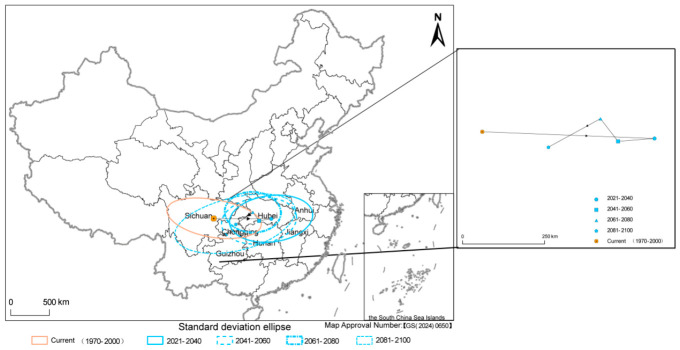
Standard deviation ellipses and centroid shifts of suitable habitats for *P. americana* under the SSP3-7.0 climate scenario. The ellipses represent the spatial distribution ranges of suitable habitats during the current period (1970–2000) and future periods (2021–2040, 2041–2060, 2061–2080, and 2081–2100). The inset shows the migration trajectory of the habitat centroid across different periods.

**Figure 13:**
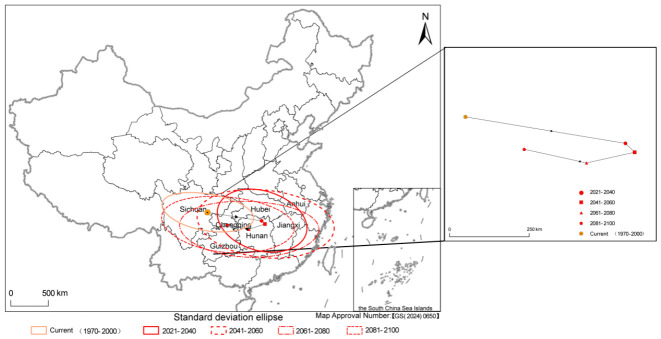
Standard deviation ellipses and centroid shifts of suitable habitats for *P. americana* under the SSP5-8.5 climate scenario. The ellipses represent the spatial distribution ranges of suitable habitats during the current period (1970–2000) and future periods (2021–2040, 2041–2060, 2061–2080, and 2081–2100). The inset shows the migration trajectory of the habitat centroid across different periods.

Centroid-shift analysis provided quantitative evidence for non-linear redistribution of suitable habitats under different climate scenarios (Figure 10, Figure 11, Figure 12 and Figure 13; Table 3). Under SSP1-2.6, the centroid showed an oscillatory expansion pattern. The displacement distance increased from 514.3 km in 2021–2040 to 817.2 km in 2041–2060, then declined to 525.6 km in 2061–2080 and increased again to 754.1 km in 2081–2100. The migration direction was mainly northeastward during 2021–2060 (93.2–101.5°), shifted northwestward in 2061–2080 (352.8°), and returned northeastward by 2081–2100 (28.7°), with a net displacement of 589.4 km. Under SSP2-4.5, centroid displacement first increased and then declined. The distance increased from 232.5 km in 2021–2040 to 461.3 km in 2041–2060, decreased by 78.2 km in 2061–2080, and finally reached 253.8 km in 2081–2100. Directionally, the centroid shifted from southeastward movement in 2021–2040 (145.2°) to northeastward movement in 2041–2060 and 2061–2080 (84.6° and 98.3°), followed by a southward shift in 2081–2100 (183.5°). Under SSP3-7.0, the centroid showed a declining displacement trend after an early peak. The maximum distance occurred in 2021–2040 (732.4 km), after which the distance decreased by 407.6 km by the end of the century. The direction remained relatively stable toward the northeast during 2021–2060 (82.1–86.3°), shifted north-northeastward in 2061–2080 (24.5°), and then turned southeastward in 2081–2100 (116.7°). This indicates that stronger forcing did not produce a simple continuous poleward displacement. Under SSP5-8.5, the centroid followed a two-phase expansion–contraction trajectory. The displacement distance increased from 742.8 km in 2021–2040 to 784.5 km in 2041–2060, then declined to 603.2 km in 2061–2080 and 300.6 km in 2081–2100. The migration direction remained southeastward during 2021–2060 (104.3–110.7°), shifted further south-southeastward in 2061–2080 (137.5°), and turned southwestward by 2081–2100 (201.8°), producing a non-linear spatial loop with a net displacement of 442.2 km.

Overall, these centroid results confirm that future redistribution of *P. americana* suitable habitats is scenario-dependent and non-monotonic. Low and moderate forcing scenarios mainly produced northeastward or oscillatory shifts, whereas stronger forcing scenarios resulted in directional deflection, contraction of centroid displacement, or loop-like trajectories. Therefore, the projected changes should be interpreted as spatial reorganization of climatic suitability rather than as a simple poleward range expansion.

## 4. Discussion

Model accuracy and robustness.

Filtering base learners by TSS (>0.70) and excluding the underperforming SRE yielded a BIOMOD2 ensemble (EM) with markedly higher skill than any single algorithm (TSS = 0.804, 95% CI: 0.783–0.825; AUC = 0.967, 95% CI: 0.959–0.975). This accords with comparative SDM studies showing that performance-filtered, performance-weighted ensembles temper algorithm-specific bias and improve transferability relative to MaxEnt-only or single-model approaches [23,32,33]. The strong discrimination indicates a stable, learnable relationship between *P*. *americana* occurrences and underlying environments, providing confidence for interpretation and projection [34].

Climatic controls on suitability: a two-gate mechanism plus variability.

Normalized importance synthesis consistently identified May shortwave radiation (Srad05), January mean temperature (Tavg01), and temperature annual range (BIO07) as dominant predictors, with October radiation (Srad10), October precipitation (Prec10), and August radiation (Srad08) contributing secondarily. Together they outline a mechanistic scheme in which winter minima define the outer envelope of persistence (survival gate), spring energy regulates the tempo of early growth and competitive pre-emption (energy gate), and intra-annual thermal amplitude filters phenological plasticity and stress tolerance [35,36]. Prior invasive-plant SDMs often emphasize winter temperature and growing-season moisture, whereas our results suggest that spring radiation and annual thermal amplitude also play important roles in shaping the potential distribution of *P. americana*. This interpretation is consistent with recent experimental evidence showing that invasive plants may maintain stronger growth, photosynthetic capacity, and antioxidant defense under nutrient stress than related native species. For example, Dai et al. [37] reported that invasive *Wedelia trilobata* showed higher biomass accumulation, chlorophyll content, and antioxidant enzyme activity than native W. chinensis under low-nitrogen conditions. Although this evidence comes from another invasive species, it supports the broader view that physiological stress tolerance may help invasive plants persist in heterogeneous or marginal habitats. Therefore, the importance of Srad05, Tavg01, and BIO07 in our model may reflect not only broad climatic constraints but also the environmental conditions under which stress-tolerant establishment and seasonal growth are more likely to occur.

Spatial redistribution of habitat quality under climate forcing.

Across CMIP6 scenarios, suitability does not rise uniformly. As winter minima relax and spring radiation approaches physiological optima, moderate-suitability belts expand northward and upslope; however, high-suitability patches tend to fragment under stronger forcing, indicating a quality–quantity decoupling driven by hydroclimatic variability and extremes [38,39]. This reconciles reports of range expansion with field observations of heterogeneous invasion pressure and implies that risk will concentrate where climatic opportunity intersects propagule pathways—transport verges, river corridors, and cropland–forest ecotones—rather than as a continuous front [40,41]. The projected increase in low- and moderate-suitability areas should therefore be interpreted cautiously. Such areas may represent climatically permissive but ecologically variable habitats, where local nutrient status, disturbance intensity, soil conditions, and propagule pressure could determine whether potential suitability is translated into actual invasion. This distinction is important because recent physiological studies indicate that invasive plants may perform well under resource-limited conditions, but realized establishment still depends on local habitat context and management disturbance.

Centroid dynamics: non-monotonic and pathway-dependent.

Centroid analyses reveal non-linear, pathway-specific trajectories rather than steady poleward drift. Under low forcing (SSP1-2.6), an oscillate–expand pattern emerges (early NE advance, mid-century retraction, late-century recovery). Under moderate forcing (SSP2-4.5), the dominant axis reorients in phases (SE → NE → S), consistent with repeated resetting by seasonal energy and winter thresholds. High forcing (SSP3-7.0) shows an early displacement peak followed by damping and late pivot toward E/SE, while SSP5-8.5 traces an expansion–contraction loop (ESE → SSE → SSW). Compared with studies reporting quasi-linear northward drift of other invaders [42,43], these reversals underscore the role of interannual variability and extremes in steering range centers [44,45], again consistent with our “two-gate + variability” framework.

Contributions relative to existing work and practical implications.

Methodologically, this study moves beyond MaxEnt-centric practice by integrating multiple learners under a common data architecture, filtering by discrimination thresholds, and weighting by performance to yield consensus and agreement layers [32,34]. Substantively, it advances the field by identifying specific climatic bands—spring shortwave radiation, winter mean temperature, and thermal amplitude—as coordinated drivers that mechanistically link climate to invasion dynamics [35,36]. Theoretically, we extend niche-based invasion models by formalizing a two-gate mechanism superimposed on a variability axis and by positing a testable trade-off: geographic opportunity can expand while the spatial cohesion of optimal habitat declines under strong forcing [41,46].

Management implications and future research.

Practically, our outputs support a three-level management strategy for *P. americana*. First, surveillance should prioritize current high-suitability cores in eastern and southern China and emerging marginal zones in northern, northwestern, and elevation-limited regions. Second, pathway management should focus on road–rail corridors, river valleys, forest margins, disturbed verges, and cropland–forest ecotones, where climatic opportunity and propagule pressure may overlap. Third, habitat-level management should reduce establishment opportunities in disturbed sites through early removal, vegetation restoration, and prevention of seed dispersal after control operations. Recent work also suggests that invasive plant biomass may have potential value after safe processing. For instance, Gul et al. [47] showed that biochar derived from the invasive weed *Parthenium hysterophorus*, especially when combined with urea, improved soil properties, enhanced wheat growth under Cd stress, and reduced Cd transfer to plant tissues. Although this strategy cannot be directly transferred to *P. americana* without species-specific safety evaluation, it highlights a possible “removal–safe disposal–resource utilization” pathway for invasive-plant management. Future studies should therefore integrate climatic suitability, land use, soil conditions, propagule pressure, and post-removal biomass treatment to better distinguish potential suitability from realized invasion risk [24].

## 5. Conclusions

Using a performance-filtered BIOMOD2 ensemble, this study offers a high-accuracy assessment of the climatic drivers and future distribution of *P*. *americana* in China. Three coordinated variables—May shortwave radiation, January mean temperature, and annual temperature range—emerged as dominant controls, jointly shaping early-season growth, overwinter survival, and tolerance to thermal variability. These mechanisms explain both the species’ current concentration in warm–humid eastern China and its projected expansion into northern and higher-elevation regions as climates warm. Future scenarios indicate that while geographic opportunity increases, high-quality habitats may fragment under stronger forcing, producing non-linear, pathway-specific centroid shifts rather than a simple poleward trend. This highlights the central role of climatic variability and extreme events in invasion dynamics. Our findings refine climate-driven invasion theory by integrating physiological mechanisms into distribution forecasts and provide actionable guidance for surveillance timing, pathway management, and habitat interventions aimed at limiting spring energy availability.

## Figures and Tables

**Table 1:** Environmental variables considered for *P. americana* distribution modelling.

Variable	Description	Unit
BIO01	Annual Mean Temperature	°C
BIO02	Mean Diurnal Range	°C
BIO03	Isothermality (BIO2/BIO7) (×100)	-
BIO04	Temperature Seasonality	-
BIO05	Max Temperature of Warmest Month	°C
BIO06	Min Temperature of Coldest Month	°C
BIO07	Temperature Annual Range (BIO5–BIO6)	°C
BIO08	Mean Temperature of Wettest Quarter	°C
BIO09	Mean Temperature of Driest Quarter	°C
BIO10	Mean Temperature of Warmest Quarter	°C
BIO11	Mean Temperature of Coldest Quarter	°C
BIO12	Annual Precipitation	mm
BIO13	Precipitation of Wettest Month	mm
BIO14	Precipitation of Driest Month	mm
BIO15	Precipitation Seasonality	mm
BIO16	Precipitation of the Wettest Quarter	mm
BIO17	Precipitation of the Driest quarter	mm
BIO18	Precipitation of Warmest Quarter	mm
BIO19	Precipitation of Coldest Quarter	mm
Elev	Elevation	m
Prec01-12	Monthly precipitation from January to December	mm
Srad01-12	Monthly solar radiation from January to December	kJ/(m^2^ d)
Wind01-12	Monthly wind speed from January to December	m/s
Vapr01-12	Monthly vapor pressure from January to December	kPa
Tmin01-12	Monthly minimum temperature from January to December	°C
Tmax01-12	Monthly maximum temperature from January to December	°C
Tavg01-12	Monthly mean temperature from January to December	°C

**Table 2:** Criteria used to evaluate model performance based on AUC, TSS, and Kappa.

Indicator	Excellent	Good	Fair	Poor	Fail
AUC	1.00–0.90	0.90–0.80	0.80–0.70	0.70–0.60	0.50–0.00
TSS	1–0.85	0.85–0.70	0.70–0.55	0.55–0.4	0.40–0.00
Kappa	1–0.85	0.85–0.70	0.70–0.55	0.55–0.4	0.40–0.00

**Table 3:** Centroid displacement distance and direction of suitable habitats for *P. americana* under future climate scenarios.

Scenario	Period	Displacement Distance (km)	Direction/Azimuth (°)	Main Direction
SSP1-2.6	2021–2040	514.3	93.2	East-northeast
SSP1-2.6	2041–2060	817.2	101.5	East-northeast
SSP1-2.6	2061–2080	525.6	352.8	Northwest/north-northwest
SSP1-2.6	2081–2100	754.1	28.7	Northeast
SSP2-4.5	2021–2040	232.5	145.2	Southeast
SSP2-4.5	2041–2060	461.3	84.6	East-northeast
SSP2-4.5	2061–2080	383.1	98.3	East
SSP2-4.5	2081–2100	253.8	183.5	South
SSP3-7.0	2021–2040	732.4	82.1	East-northeast
SSP3-7.0	2041–2060	638.3	86.3	East-northeast
SSP3-7.0	2061–2080	549.8	24.5	Northeast
SSP3-7.0	2081–2100	324.8	116.7	Southeast
SSP5-8.5	2021–2040	742.8	104.3	East-southeast
SSP5-8.5	2041–2060	784.5	110.7	East-southeast
SSP5-8.5	2061–2080	603.2	137.5	Southeast
SSP5-8.5	2081–2100	300.6	201.8	Southwest

Direction is expressed as degrees clockwise from north.

## Data Availability

All the required data are uploaded as Supplementary Materials.

## Supplementary Materials

The following supporting information can be downloaded at: https://www.mdpi.com/article/10.3390/plants15111747/s1, Figure S1: Comparison of predictive performance among nine BIOMOD2 algorithms for *P. americana* distribution modelling.

## References

[1] Walther G.-R., Roques A., Hulme P.E., Sykes M.T., Pyšek P., Kühn I., Zobel M., Bacher S., Botta-Dukát Z., Bugmann H. (2009). Alien species in a warmer world: Risks and opportunities. Trends Ecol. Evol..

[2] Bellard C., Bertelsmeier C., Leadley P., Thuiller W., Courchamp F. (2012). Impacts of climate change on the future of biodiversity. Ecol. Lett..

[3] Early R., Bradley B.A., Dukes J.S., Lawler J.J., Olden J.D., Blumenthal D.M., Gonzalez P., Grosholz E.D., Ibañez I., Miller L.P. (2016). Global threats from invasive alien species in the twenty-first century and national response capacities. Nat. Commun..

[4] Pyšek P., Hulme P.E., Simberloff D., Bacher S., Blackburn T.M., Carlton J.T., Dawson W., Essl F., Foxcroft L.C., Genovesi P. (2020). Scientists’ warning on invasive alien species. Biol. Rev..

[5] Bradley B.A., Beaury E.M., Gallardo B., Ibáñez I., Jarnevich C.S., Morelli T.L., Sofaer H.R., Sorte C.J.B., Vilà M. (2024). Observed and potential range shifts of native and non-native species with climate change. Annu. Rev. Ecol. Evol. Syst..

[6] Roy H.E., Pauchard A., Stoett P., Renard Truong T., Meyerson L.A., Bacher S., Galil B.S., Hulme P.E., Ikeda T., McGeoch M.A. (2024). Curbing the major and growing threats from invasive alien species is urgent and achievable. Nat. Ecol. Evol..

[7] Flickinger H.D., Dukes J.S. (2024). A review of theory: Comparing invasion ecology and climate change-induced range shifting. Glob. Change Biol..

[8] Wallingford P.D., Morelli T.L., Allen J.M., Beaury E.M., Blumenthal D.M., Bradley B.A., Dukes J.S., Early R., Fusco E.J., Goldberg D.E. (2020). Adjusting the lens of invasion biology to focus on the impacts of climate-driven range shifts. Nat. Clim. Change.

[9] Vilà M., Espinar J.L., Hejda M., Hulme P.E., Jarošík V., Maron J.L., Pergl J., Schaffner U., Sun Y., Pyšek P. (2011). Ecological impacts of invasive alien plants: A meta-analysis of their effects on species, communities and ecosystems. Ecol. Lett..

[10] Seebens H., Blackburn T.M., Dyer E.E., Genovesi P., Hulme P.E., Jeschke J.M., Pagad S., Pyšek P., Winter M., Arianoutsou M. (2017). No saturation in the accumulation of alien species worldwide. Nat. Commun..

[11] Chen P., Shen C., Tao Z., Qin W., Huang W., Siemann E. (2024). Deterministic responses of biodiversity to climate change through exotic species invasions. Nat. Plants.

[12] Robeck P., Essl F., van Kleunen M., Pyšek P., Pergl J., Weigelt P., Mesgaran M.B. (2024). Invading plants remain undetected in a lag phase while they explore suitable climates. Nat. Ecol. Evol..

[13] Diagne C., Leroy B., Vaissière A.-C., Gozlan R.E., Roiz D., Jarić I., Salles J.-M., Bradshaw C.J.A., Courchamp F. (2021). High and rising economic costs of biological invasions worldwide. Nature.

[14] Seebens H., Bacher S., Blackburn T.M., Capinha C., Dawson W., Dullinger S., Genovesi P., Hulme P.E., van Kleunen M., Kühn I. (2021). Projecting the continental accumulation of alien species through to 2050. Glob. Change Biol..

[15] Xu Y., Ye X., Yang Q., Weng H., Liu Y., Ahmad S., Zhang G., Huang Q., Zhang T., Liu B. (2023). Ecological niche shifts affect the potential invasive risk of *Phytolacca americana* (Phytolaccaceae) in China. Ecol. Process..

[16] Nan Q., Li C., Li X., Zheng D., Li Z., Zhao L. (2024). Modeling the potential distribution patterns of the invasive plant species *Phytolacca americana* in China in response to climate change. Plants.

[17] Elith J., Leathwick J.R. (2009). Species distribution models: Ecological explanation and prediction across space and time. Annu. Rev. Ecol. Evol. Syst..

[18] Davies S.C., Thompson P.L., Gomez C., Nephin J., Knudby A., Park A.E., Friesen S.K., Pollock L.J., Rubidge E.M., Anderson S.C. (2023). Addressing uncertainty when projecting marine species’ distributions under climate change. Ecography.

[19] Davis A.J.S., Groom Q., Adriaens T., Vanderhoeven S., De Troch R., Oldoni D., Desmet P., Reyserhove L., Lens L., Strubbe D. (2024). Reproducible WiSDM: A workflow for reproducible invasive alien species risk maps under climate change scenarios using standardized open data. Front. Ecol. Evol..

[20] Koldasbayeva D., Zaytsev A. (2025). Foundation for unbiased cross-validation of spatio-temporal models for species distribution modeling. Ecol. Inform..

[21] Araújo M.B., New M. (2007). Ensemble forecasting of species distributions. Trends Ecol. Evol..

[22] Thuiller W., Lafourcade B., Engler R., Araújo M.B. (2009). BIOMOD—A platform for ensemble forecasting of species distributions. Ecography.

[23] Hao T., Elith J., Guillera-Arroita G., Lahoz-Monfort J.J. (2020). A review of evidence about use and performance of species distribution modelling ensembles like BIOMOD. Divers. Distrib..

[24] Frans V.F., Liu J. (2024). Gaps and opportunities in modelling human influence on species distributions in the Anthropocene. Nat. Ecol. Evol..

[25] Xu H., Qiang S., Genovesi P., Ding H., Wu J., Meng L., Han Z., Miao J., Hu B., Guo J. (2012). An inventory of invasive alien species in China. NeoBiota.

[26] Liu C., Wolter C., Xian W., Jeschke J.M. (2020). Species distribution models have limited spatial transferability for invasive species. Ecol. Lett..

[27] Roberts D.R., Bahn V., Ciuti S., Boyce M.S., Elith J., Guillera-Arroita G., Hauenstein S., Lahoz-Monfort J.J., Schröder B., Thuiller W. (2017). Cross-validation strategies for data with temporal, spatial, hierarchical, or phylogenetic structure. Ecography.

[28] Fick S.E., Hijmans R.J. (2017). WorldClim 2: New 1-km spatial resolution climate surfaces for global land areas. Int. J. Climatol..

[29] Eyring V., Bony S., Meehl G.A., Senior C.A., Stevens B., Stouffer R.J., Taylor K.E. (2016). Overview of the Coupled Model Intercomparison Project Phase 6 (CMIP6). Geosci. Model Dev..

[30] O’Neill B.C., Kriegler E., Riahi K., Ebi K.L., Hallegatte S., Carter T.R., Mathur R., van Vuuren D.P. (2016). The roads ahead: Narratives for shared socioeconomic pathways describing world futures in the 21st century. Glob. Environ. Change.

[31] Thuiller W., Georges D., Engler R., Breiner F. (2023). biomod2: Ensemble Platform for Species Distribution Modeling, R package version 4.2-2.

[32] Araújo M.B., Anderson R.P., Márcia Barbosa A., Beale C.M., Dormann C.F., Early R., Garcia R.A., Guisan A., Maiorano L., Naimi B. (2019). Standards for distribution models in biodiversity assessments. Sci. Adv..

[33] Norberg A., Abrego N., Blanchet F.G., Adler F.R., Anderson B.J., Anttila J., Araújo M.B., Dallas T., Dunson D., Elith J. (2019). A comprehensive evaluation of predictive performance of 33 species distribution models at species and community levels. Ecol. Monogr..

[34] Zurell D., Franklin J., König C., Bouchet P.J., Dormann C.F., Elith J., Fandos G., Feng X., Guillera-Arroita G., Guisan A. (2020). A standard protocol for reporting species distribution models. Ecography.

[35] Buckley L.B., Kingsolver J.G. (2019). Environmental variability shapes evolution, plasticity and species’ distributions. Ecol. Lett..

[36] Kearney M.R., Porter W.P. (2020). NicheMapR—An R package for biophysical modelling: The ectotherm and Dynamic Energy Budget models. Ecography.

[37] Dai Z.-C., Kong F.-L., Li Y.-F., Ullah R., Ali E.A., Gul F., Du D.-L., Zhang Y.-F., Jia H., Khan I.U. (2024). Strong invasive mechanism of Wedelia trilobata via growth and physiological traits under nitrogen stress condition. Plants.

[38] Fischer E.M., Sippel S., Knutti R. (2021). Increasing probability of record-shattering climate extremes. Nat. Clim. Change.

[39] Thompson L.M., Powers S.D., Appolon A., Hafker P., Milner L., Parry D., Agosta S.J., Grayson K.L. (2021). Climate-related geographical variation in performance traits across the invasion front of a widespread non-native insect. J. Biogeogr..

[40] Seebens H., Blackburn T.M., Dyer E.E., Genovesi P., Hulme P.E., Jeschke J.M., Pagad S., Pyšek P., van Kleunen M., Winter M. (2018). Global rise in emerging alien species results from increased accessibility of new source pools. Proc. Natl. Acad. Sci. USA.

[41] Catford J.A., Bode M., Tilman D. (2022). Contextual factors determine the impact of alien species on ecosystems. Ecol. Lett..

[42] Chen I.-C., Hill J.K., Ohlemüller R., Roy D.B., Thomas C.D. (2011). Rapid range shifts of species associated with high levels of climate warming. Science.

[43] Parmesan C., Yohe G. (2003). A globally coherent fingerprint of climate change impacts across natural systems. Nature.

[44] Vázquez D.P., Gianoli E., Morris W.F., Bozinovic F. (2017). Ecological and evolutionary impacts of changing climatic variability. Biol. Rev..

[45] Trisos C.H., Merow C., Pigot A.L. (2020). The projected timing of abrupt ecological disruption from climate change. Nature.

[46] García-Valdés R., Gotelli N.J., Zavala M.A., Purves D.W., Araújo M.B. (2015). Effects of climate, species interactions, and dispersal on decadal colonization and extinction rates of Iberian tree species. Ecol. Model..

[47] Gul F., Khan I.U., Li G., Ullah R., Ibrahim M.A., Ullah K., Khan Z., Du D. (2024). Co-application of Parthenium biochar and urea effectively mitigate cadmium toxicity during wheat growth. Ecotoxicol. Environ. Saf..

