# Supplementary material for: Climate-Driven Prediction of the Future Distribution of Phytolacca americana L. Using a BIOMOD2 Ensemble Modelling Framework
Source: Plants (Basel). 2026 Jun 4;15(11):1747. doi: 10.3390/plants15111747 (PMC13258892; doi:10.3390/plants15111747)
Supplement: Supplementary file 1 [file plants-15-01747-s001.zip › plants-4252406-supplementary.pdf]

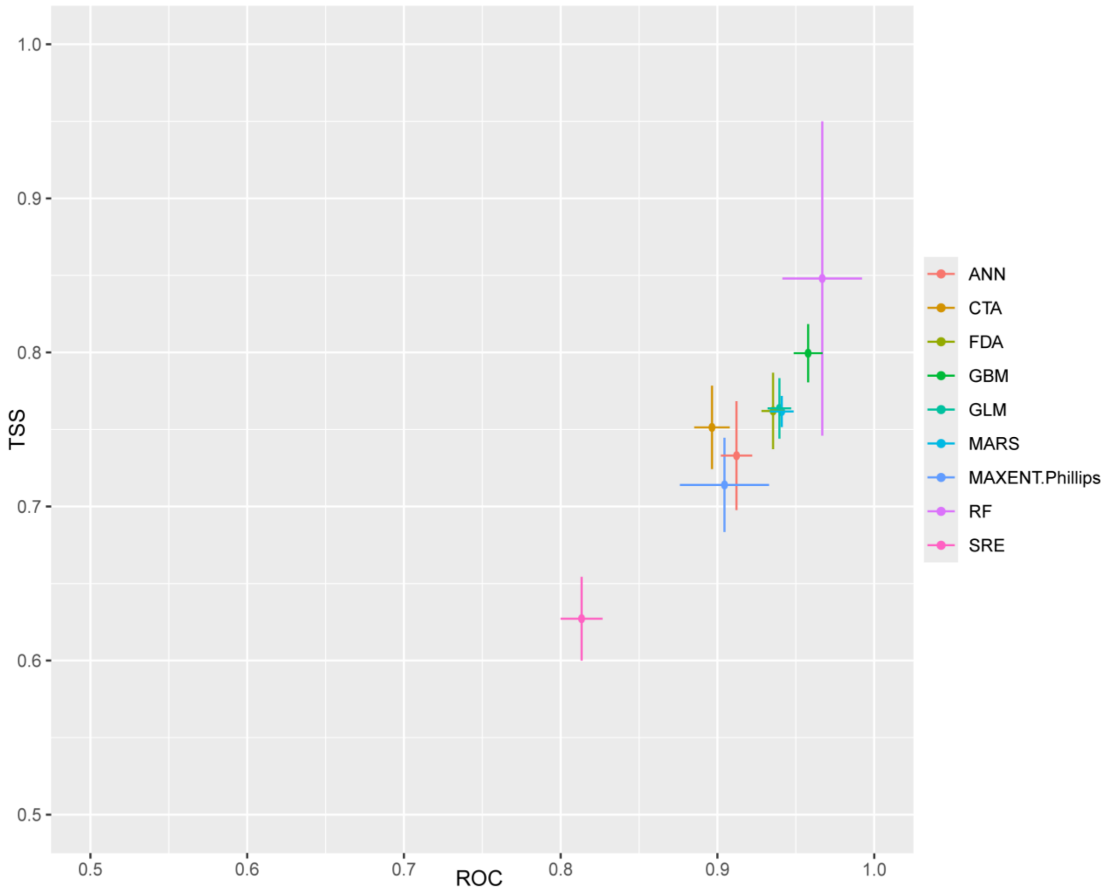

S1. Comparison of predictive performance among nine BIOMOD2 algorithms for *P. americana* distribution modelling. The x-axis indicates ROC values and the y-axis indicates TSS values. Points show mean model performance, and error bars represent the corresponding standard deviations across repeated runs. Algorithms with higher ROC and TSS values were retained as better-performing models for subsequent ensemble modelling.
